# Experiences and perceptions of COVID-19 infection and vaccination among Palestinian refugees in Jerash camp and Jordanian citizens: a comparative cross-sectional study by face-to-face interviews

**DOI:** 10.1186/s40249-022-01047-y

**Published:** 2022-12-13

**Authors:** Mohammad A. I. Al-Hatamleh, Ma’mon M. Hatmal, Sulaf H. F. Mustafa, Mohammad Alzu’bi, Ali F. AlSou’b, Shahed N. S. Abughanam, Amin N. Olaimat, Elham T. Kateeb, Rohimah Mohamud

**Affiliations:** 1 Department of Immunology, School of Medical Sciences, Universiti Sains Malaysia, Kota Bharu, 16150 Malaysia; 2 Department of Medical Laboratory Sciences, Faculty of Applied Medical Sciences, The Hashemite University, P.O. Box 330127, 13133 Zarqa, Jordan; 3 Faculty of Medicine, The University of Jordan, 11942 Amman, Jordan; 4 Faculty of Medicine, The Hashemite University, 13133 P.O. Box 330127, Zarqa, Jordan; 5 Faculty of Medicine, Al-Balqa Applied University, 19117 Al-Salt, Jordan; 6 Faculty of Medicine, Mutah University, 61710 Karak, Jordan; 7 Department of Clinical Nutrition and Dietetics, Faculty of Applied Medical Sciences, The Hashemite University, P.O. Box 330127, 13133 Zarqa, Jordan; 8 Oral Health Research and Promotion Unit, Faculty of Dentistry, Al-Quds University, P.O. Box 51000 Jerusalem, Palestine

**Keywords:** SARS-CoV-2, Vaccine rollout, Health equity, Refugee vaccination, Vaccine hesitancy, Adverse effects

## Abstract

**Background:**

During the COVID-19 vaccination, the access to vaccines has been unequal among countries and individuals, for example low-income countries displayed significant low levels of vaccination. Furthermore, most refugees are living in developing low-income countries which struggling to access the essential health-care services including vaccination. Thus, the objective of this study was to assess the experiences and perceptions of COVID-19 infection and vaccination among Palestine refugees in Jerash camp compared to resident Jordanian citizens.

**Methods:**

A face-to-face interview-based comparative cross-sectional study was carried out among Palestine refugees in Jerash camp located in northern Jordan and Jordanian citizens from different cities in Jordan from October, 2021 to March, 2022. A Chi-square test was used to determine the differences in the experiences and perceptions of COVID-19 infection and vaccination between Palestinian refugees and resident Jordanian citizens. Logistic regression analysis was performed to predict factors associated with the beliefs, barriers and hesitancy towards COVID-19 vaccines.

**Results:**

The total number of participants was 992, with 501 (50.5%) Palestinian refugees and 491 (49.5%) Jordanian citizens. Most participants (64.1%) who have never been tested for COVID-19 were from the refugees (*P* < 0.001), whereas about 80.3% of the participants tested for COVID-19 at private healthcare institutions were citizens (*P* < 0.001). While 70.0% of the participants who tested positive for COVID-19 (*n* = 303) were from the refugees (*P* < 0.001). Compared to the citizens, the refugees had significantly lower levels of beliefs about the safety (*P* = 0.008) and efficiency (*P* < 0.001) of COVID-19 vaccines. They also had lower rates of vaccine hesitancy (*P* = 0.002) and vaccine uptake (*P* < 0.001), and a higher rate of facing difficulties during registration for COVID-19 vaccination (*P* < 0.001). Furthermore, refugees have more negative attitudes toward the importance and implementation of COVID-19 precautionary activities, including wearing face masks, practicing social distancing and following proper prevention hygiene compared to citizens (*P* < 0.001). The regression analysis showed that gender (*P* < 0.001), age (*P* < 0.001) and level of education (*P* = 0.001) were significantly associated with COVID-19 vaccine hesitancy. Also, being a refugee (*P* < 0.001) and being a male (*P* = 0.012) were significantly associated with facing more difficulties upon the registration to receive a COVID-19 vaccine.

**Conclusions:**

This study showed that, compared to citizens, refugees had lower attitudes and practices toward COVID-19 infection and vaccination. They also had and a lower rate of COVID-19 vaccine hesitancy and uptake with limited access to vaccines. Government sectors and non-government organizations should implement policies and regulations to raise the awareness of refugees towards COVID-19 infection, testing, preventive measures, and the safety and efficacy of vaccines.

**Supplementary Information:**

The online version contains supplementary material available at 10.1186/s40249-022-01047-y.

## Background

On March 11, 2020, the World Health Organization (WHO) announced the coronavirus disease 2019 (COVID-19) outbreak, caused by the severe acute respiratory syndrome coronavirus 2 (SARS-CoV-2), as a global pandemic [1]. The main clinical symptoms of COVID-19 include cough, sore throat, headache, fatigue, fever, myalgia and breathlessness, as well as conjunctivitis [2]. While the severity of symptoms in COVID-19 patients is rapidly progressive and can be at a severe level, most cases display mild to moderate symptoms, which make it similar to seasonal conventional flu [3].

The COVID-19 pandemic has challenged healthcare systems worldwide and stressed the global economy [4]. It had also led to the first rise in extreme poverty in two decades and increased the in between-country economic and health inequalities to the truly shocking levels [5]. The COVID-19 pandemic has restricted the provision and the accessibility of healthcare services. Regardless of region, all countries have had to adapt their healthcare systems to combat the pandemic with minimal loss. While high-income countries (HICs) which have a coordinated network of well-prepared healthcare facilities have experienced a considerable burden of COVID-19 infection, it has hit low-income countries (LICs) the hardest as they are already bearing the brunt of the global disaster. Not surprisingly, a recent report showed that since the WHO declared the pandemic, the total number of COVID-19 deaths has been four times higher in LICs than HICs [6]. Even before the COVID-19 pandemic, inequality in access to healthcare services was a major challenge in low- and middle-income countries [7]. Amid the pandemic, these countries experienced significant barriers related to lack of testing available, protective personal equipment, advanced healthcare services, as well as the global vaccine supply inequities [8, 9]. Therefore, the pandemic exacerbating the challenges of health inequities in poor communities resulting in new waves of negative socio-economic consequences.

To combat the pandemic, the world has witnessed exceptional efforts to quickly and safely develop vaccines against the COVID-19 using multiple platforms, including RNA, DNA, viral vectors, recombinant protein, inactivated and live attenuated [10]. Currently, ten vaccines (i.e., Pfizer-BioNTech, Moderna, AstraZeneca-Oxford, Johnson & Johnson, Sinopharm, Sinovac, COVAXIN, Covovax, Nuvaxovid and CanSino) are authorized and made available for immunization against COVID-19 as they showed adequate results in the clinical trials with high efficacy rates [11]. As of July 20, 2022, 12.23 billion doses have been administrated and 66.8% of the people on the planet have received their first dose of a COVID-19 vaccine [12]. However, this percentage is varied by country. In LICs, one in five people has been vaccinated at least one dose (20.1%) compared to three in four people (72.1%) in HICs. This variation is mainly attributed to the fact that HICs started the vaccination process two months earlier than LICs [13]. In addition, LICs are still facing challenges in the COVID-19 vaccine procurement and rollout, as well as the high levels of public knowledge, attitude and acceptance towards COVID-19 vaccines [14, 15].

Several organizations, like the COVID-19 Vaccine Global Access (COVAX), were established for facilitating quick, fair, prioritize, global delivery and access to COVID-19 vaccines [14]. Due to government’s insufficient vaccine access funds, modest donor funds and donated vaccine doses, most LICs and middle-income countries (MICs) relied on the COVAX to obtain COVID-19 vaccines [16]. Although these countries were prioritizing healthcare workers in their vaccination programs, the lack of supply was limiting their efforts, and thus healthcare workers waited longer than they expected before they receive COVID-19 vaccines. In addition, the trade disputes, lack of global pricing policies and impediments of local vaccine manufacturing have also contributed in hindering vaccine access in LICs and MICs [17].

Although all countries are affected by the COVID-19 pandemic, the burden is not shared equally. Countries hosting large numbers of refugees face harder challenges such as providing COVID-19 vaccination, as well as diagnostic and treatment tools, knowing that LICs and MICs have most of the world’s refugees [18]. As the second host country of refugees per capita globally [19], Jordan faces several challenges in mounting an adequate response to the COVID-19 pandemic. Jordan hosts refugees of Palestinian, Syrian, Iraqi, Yemeni, Sudanese, Somali and other nationalities [20]. The majority of refugees in Jordan are Palestinians; the country hosts more than 2 million registered Palestinian refugees [21]. More than 17% of the registered Palestinian refugees in Jordan are accommodated in ten official camps [21]. Jerash camp had the highest prevalence of poverty with the lowest income level of all Palestinian refugee camps [22]. A recent report by the United Nations International Children’s Emergency Fund (UNICEF) showed that 52.7% of refugees in Jerash camp are living below the national poverty line [23]. Unlike the situations of other Palestinian refugee camps, the Palestinian refugees and their descendants who live in Jerash camp are still without citizenships, although they came to this camp since 1967. One of the major challenges in this camp is the inadequate healthcare facilities with lack of health insurance and coverage, as well as the high prevalence of chronic health problems among refugees [24].

Jordan government started a national campaign directed toward the awareness in early time of the pandemic, which was followed by community-wide strict measures to suppress the public health effects of COVID-19 [25]. Along with the Jordanian citizens, Jordan government invited everyone who is residing in the country to get vaccinated against COVID-19 freely and regardless their residency status. Thus, Jordan was among the world’s first country to start COVID-19 vaccinations for refugees [26]. However, amid the huge number of studies on the experiences and perceptions of COVID-19 infection and vaccination, reports that focus on refugees (especially those who live in camps) are currently lacking, not just in Jordan but also abroad. As they represent a significant proportion of the Jordan population, refugees must not be neglected in the COVID-19 pandemic in Jordan and globally. Therefore, this study assessed the experiences and perceptions of COVID-19 infection and vaccination among Palestine refugees in Jerash camp compared to resident Jordanian citizens.

## Methods

### Study design and settings

A face-to-face interview-based comparative cross-sectional study was carried out in Jerash camp which is located in Jerash governorate in northern Jordan from October 16, 2021, to March 22, 2022. Jerash camp (also known as Gaza camp) is an emergency camp was established in 1968 for Palestinian refugees who came from the Gaza Strip during the Arab-Israeli war. Jerash camp is officially recognized by The United Nations Relief and Works Agency for Palestine Refugees in the Near East (UNRWA) and today it hosts approximately more than 31,000 registered refugees [23].

### Ethical considerations

This study was conducted according to the principles expressed in the Declaration of Helsinki. The study was approved by the Institutional Review Board (IRB) committee of The Hashemite University (protocol code: 3/10/2020/2021). After giving the participants sufficient information about the study, verbal consent was obtained from the participants prior to their enrolling in this study.

### Participants and sample size

All the participated refugees are living and working in Jerash camp only. The citizens who participated in this study were from different cities in Jordan and they have not experienced the life in a refugee camp. The current study included only adults aged ≥ 18 years. The participants were randomly selected from the general populations through street interviews. Sample size of refugees was determined using Raosoft online sample size calculator. The calculation was based on 5% margin of error, 95% confidence interval and 50% response distribution. The minimum representative sample size was 380 refugees. To ensure accuracy, the sample size was increased to 501 refugees, and nearly similar number of citizens was recruited to participate in this study.

### Data collection

The data collection was conducted in face-to-face interviews. Supervised by senior researchers, a group of trained medical students used a standardized interview protocol to record participants’ responses to a pre-tested questionnaire. To ensure the quality of face-to-face interviews, a few tips have been adapted from Jacob and Furgerson, 2012 [27] and followed into the design of the interview protocol. The interviewers introduced the research team members and their affiliations, explained the aim of the study, assured the confidentiality and anonymity of participants’ data during and after the information have been collected, and recruited only participants who fulfilled the inclusion criteria and consented to participate in the study.

### Survey tool

Since Arabic is the official language in Jordan, the Arabic version of a well-validated questionnaire used for evaluation of the adverse effects and perceptions following COVID-19 vaccination in Jordan [28] and the Arab World [29]. The questionnaire was modified to fit the population context and the aim of this study [Additional file 1: Survey tool (English version)]. Then, for the aim of validation, a panel of expert and independent researchers from different research areas, including epidemiology, community health nursing, occupational safety and health, public health nutrition, immunology, microbiology and infectious diseases, were invited to review the questionnaire, and they provided positive feedback with minor comments. Furthermore, a pilot study was carried out on 40 participants from refugees and citizens to evaluate the comprehensibility and clarity of the questionnaire (this sample was excluded from the formal evaluation). The internal consistency (reliability) of the questionnaire was estimated using Cronbach’s alpha test and the test value was equal to 0.81, which is considered high, above the cut-off limit (0.70).

### Statistical analysis

Data were analyzed for frequencies and percentages which were used as descriptive statistics. A Chi-square test was used to determine whether there are statistically significant differences between refugees and citizens in their sociodemographic characteristics, access to healthcare services, health insurance coverage, COVID-19 infection and vaccination rates, COVID-19 testing, COVID-19 symptoms, vaccine hesitancy and adverse effects following vaccination, as well as their attitudes and beliefs towards COVID-19 infection, vaccination and related measures. Also, it was used to compare the post-vaccination adverse effects among all the participants based on the type of vaccine and the number of doses as secondary outcomes. Moreover, logistic regression analysis was performed to identify significant factors associated with COVID-19 vaccine hesitancy and difficulties upon the registration to receive a COVID-19 vaccine, as well as the participant’s belief in the long-term safety of the COVID-19 vaccines, the effectiveness of COVID-19 vaccines in combating the pandemic, and the conspiracy theory which says that SARS-CoV-2 is a biological weapon developed at a lab as an artificial creation. Responses were coded as 1 = yes and 0 = no. In the logistic regression analysis, simple logistic regression was performed to obtain the crude odds ratio (C*OR*), and those with a *P*-value of less than 0.25 were considered important and included in the multiple logistic regression to obtain their adjusted odds ratio (A*OR*). Based on several studies, it was reported that using more traditional levels (e.g., 0.05) as *p*-value cut-off points can fail in identifying variables known to be important [30–32]. Hence, similar to recent studies [33, 34], the process of variable selection for the multiple logistic regression was based on a *p*-value of less than 0.25. The forward LR and backward LR methods were used in the multiple logistic regression, and the final model was run using the enter method to obtain the preliminary main effect model. The fit of the final model was assessed using the Hosmer-Lemeshow (H-L) test, multicollinearity (MC), the interaction between the variables, and the receiver operating characteristics (ROC) curve to obtain the area under the curve (AUC). Variance inflation factor (VIF) was used to detect the severity of MC; no MC if VIF is less than 10. Furthermore, the Mantel-Haenszel chi-square test was performed to evaluate the effects of age, gender and education level as confounding factors (Additional file 2: Mantel-Haenszel chi-square test). All statistical analyses were performed using the Statistical Package for Social Sciences (SPSS) version 26.0 (IBM Corporation, Armonk, NY, USA) and Microsoft Excel. A statistically significant difference was detected for variables at a 95% confidence interval with a *P*-value of ≤ 0.05.

## Results

### Participant characterization

The total number of participants who were involved in this study was 992; 501 (50.5%) refugees and 491 (49.5%) citizens. About half of the sample (54.2%) were females (39.5% of the refugees and 69.2% of the citizens). The age of the majority of participants (85.7%) was below 50 years old (Table 1). In terms of the number of people who studied or completed their university education and who had health insurance, there were significant differences between the refugees and citizens (*P* < 0.001); they were more in the citizens. There was also a significant difference between the refugees and the citizens in the smoking rate (*P* < 0.001); it was higher in the refugees (Table 2).

**Table 1:** The distribution of participants by gender and age

Variable	Refugee (%) (*n* = 501)	Citizen (%) (*n* = 491)	Total (%) (*n* = 992)
Gender			
Female	198 (39.5)	340 (69.2)	538 (54.2)
Male	303 (60.5)	151 (30.8)	454 (45.8)
Age (years)			
18–30	153 (30.5)	300 (61.1)	453 (45.7)
31–50	240 (47.9)	157 (32.0)	397 (40.0)
51–70	99 (19.8)	33 (6.7)	132 (13.3)
> 70	9 (1.8)	1 (0.2)	10 (1.0)

**Table 2:** Determining of statistically significant differences between refugees and citizens based on their sociodemographic characteristics (*n* = 992)

Variable	Refugee (%) (*n* = 501)	Citizen (%) (*n* = 491)		Chi–square (DF)	*P*–value
Educational level					
High school and below	385 (86.3)	61 (13.7)		419.62 (2)	< 0.001*
Undergraduate	109 (22.8)	369 (77.2)			
Postgraduate	7 (10.3)	61 (89.7)			
Health insurance					
No	398 (78.8)	107 (21.2)		329.76 (1)	< 0.001*
Yes	103 (21.1)	384 (78.9)			
Job status					
No	300 (51.2)	286 (48.8)		0.27 (1)	0.601
Yes	201 (49.5)	205 (50.5)			
Smoking status					
No	273 (43.6)	353 (56.4)		32.26 (1)	< 0.001*
Yes	228 (62.3)	138 (37.7)			
Food and/or drug allergies					
No	448 (51.6)	421 (48.4)		3.09 (1)	0.079
Yes	53 (43.1)	70 (56.9)			

Smoking includes all forms of smoking, such as cigar smoking, cigarette smoking, and pipe smoking

*DF* Degrees of freedom

*Statistically significant

### Experience of a COVID-19 infection

**Fig. 1:**
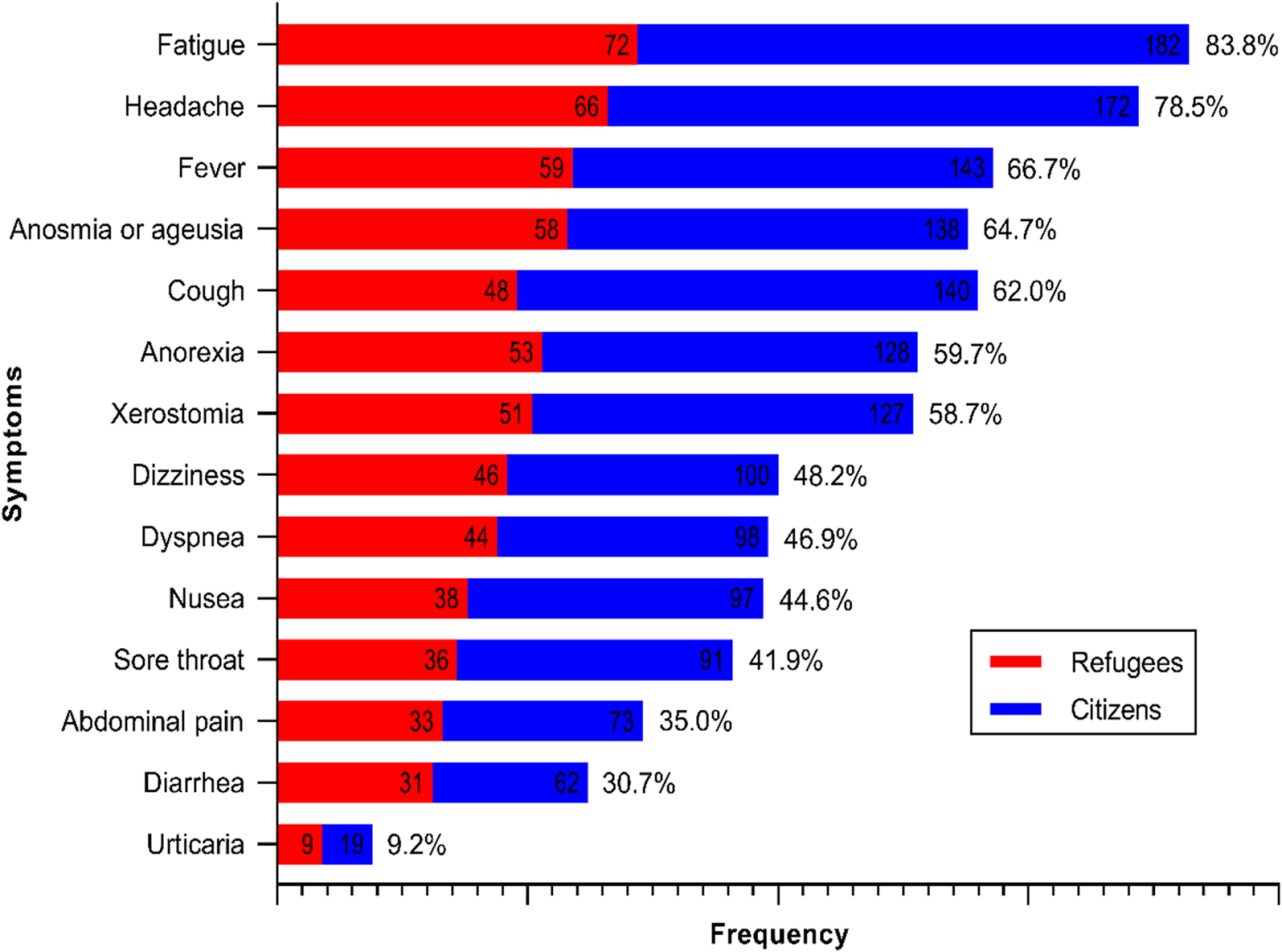
Self-reported symptoms of COVID-19 infection in the refugees and citizens (*n* = 303)

The analysis showed that the majority of participants (64.1%) who have never been tested for COVID-19 were from the refugees (*P* < 0.001). Around 80% of the participants who were capable to get a COVID-19 test at a private healthcare institution were from the citizens (*P* < 0.001). Interestingly, positive COVID-19 test was significantly lower (*P* < 0.001) among the refugees than citizens (30% vs. 70%, respectively). There was a significant difference between the refugees and citizens based on the self-reported severity of infection (*P* < 0.02). The refugees tend to experience less severe symptoms when infected with COVID-19 (Table 3). The most frequently reported symptoms were general fatigue, headache, fever, anosmia and/or ageusia, and cough (83.8%, 78.5%, 66.7%, 64.7%, and 62.0%, respectively) (Fig. 1). However, there were no significant differences between the refugees and the citizens in the frequencies of COVID-19 symptoms (*P* > 0.05).

**Table 3:** Participant experiences of COVID-19 testing and infection

Variable	Refugee (%) (*n* = 501)	Citizen (%) (*n* = 491)	Chi–square (DF)	*P*–value
Underwent a COVID–19 test				
No	223 (64.1)	125 (35.9)	39.53 (1)	< 0.001*
Yes	278 (43.2)	366 (56.8)		
Type of the healthcare facility				
Governmental or NGOs (free)	254 (48.7)	268 (51.3)	33.87 (1)	< 0.001*
Private (paid)	24 (19.7)	98 (80.3)		
Tested positive for COVID–19				
No	410 (59.5)	279 (40.5)	73.13 (1)	< 0.001*
Yes	91 (30.0)	212 (70.0)		
The severity of COVID–19 infection				
No symptoms	12 (54.5)	10 (45.5)	9.87 (3)	0.020*
Mild	18 (24.7)	55 (75.3)		
Moderate	30 (25.0)	90 (75.0)		
Severe	31 (35.2)	57 (64.8)		
The serial interval of COVID–19 symptoms				
1–2 days	6 (18.2)	27 (81.8)	7.14 (3)	0.068
3–4 days	21 (25.0)	63 (75.0)		
5–7 days	17 (24.6)	52 (75.4)		
8 days and above	37 (38.1)	60 (61.9)		
Taking antibiotics to treat COVID–19				
No	29 (23.6)	94 (76.4)	2.71 (1)	0.100
Yes	52 (32.5)	108 (67.5)		

*DF* Degrees of freedom

*Statistically significant

### Perception and experience of COVID-19 vaccination

The refugees and the citizens were significantly differed in their beliefs about the safety (*P* = 0.008) and efficiency (*P* < 0.001) of COVID-19 vaccines. Although they are facing more difficulties upon the registration to receive a COVID-19 vaccine (*P* < 0.001), the refugees had a lower rate of COVID-19 vaccine hesitancy (*P* = 0.002),(Table 4). They were also differed in the vaccination rate (*P* < 0.001), reasons for not getting a COVID-19 vaccine (*P* < 0.001), number of doses (*P* < 0.001), type of vaccination center (*P* < 0.001), experiencing post-vaccination adverse effects (*P* = 0.002), advising other people to get a COVID-19 vaccine (*P* = 0.001), and the main source of information about COVID-19 vaccines (*P* < 0.001) (Table 4).

**Table 4:** Participant experiences of COVID-19 vaccination

Variable	Refugee (%) (*n* = 501)	Citizen (%) (*n* = 491)	Chi–square (DF)	*P*–value
The belief that COVID–19 vaccines are safe in the long–term				
No	217 (55.6)	173 (44.4)	7.06 (1)	0.008*
Yes	282 (47.0)	318 (53.0)		
The belief that COVID–19 vaccines are effective and help in combating the pandemic				
No	227 (57.6)	167 (42.4)	13.61 (1)	< 0.001*
Yes	272 (45.6)	324 (54.4)		
Faced difficulties or restrictions upon the registration to receive a COVID–19 vaccine				
No	409 (47.2)	458 (52.8)	29.12 (1)	< 0.001*
Yes	90 (73.2)	33 (26.8)		
COVID–19 vaccine hesitancy				
No	249 (56.0)	196 (44.0)	9.96 (1)	0.002*
Yes	250 (45.9)	295 (54.1)		
Refused to receive a vaccine in the past				
No	379 (47.7)	416 (52.3)	12.04 (1)	0.001*
Yes	120 (61.5)	75 (38.5)		
Received a COVID–19 vaccine				
No	171 (77.4)	50 (22.6)	82.79 (1)	< 0.001*
Yes	328 (42.7)	441 (57.3)		
If not, why?				
The belief that wearing a mask, practicing social distancing and safety measures, health standards and regulations should be enough to prevent/control COVID–19	330 (42.8)	441 (57.2)	105.33 (7)	< 0.001*
Lack of information about COVID–19 vaccines and the registration for vaccination	16 (64.0)	9 (36.0)		
Just not interested without reasons	3 (100.0)	0		
Mistrust in the companies developing COVID–19 vaccines, the government, or/and healthcare providers	18 (85.7)	3 (14.3)		
Because already experienced a COVID–19 infection	41 (71.9)	16 (28.1)		
Scared of vaccine side–effects	23 (74.2)	8 (25.8)		
Other	2 (20.0)	8 (80.0)		
Type of COVID–19 vaccine				
AstraZeneca	10 (30.3)	23 (69.7)	5.58 (3)	0.134
Pfizer–BioNTech	175 (40.6)	256 (59.4)		
Sinopharm	142 (47.3)	158 (52.7)		
Other (Sinovac and Sputnik V)	1 (33.3)	2 (66.7)		
Number of doses				
One	41 (73.2)	15 (26.8)	23.06 (1)	< 0.001*
Two	287 (40.3)	426 (59.7)		
COVID–19 vaccination center				
Government hospital	65 (38.2)	105 (61.8)	28.67 (3)	< 0.001*
Government primary health care	220 (45.1)	268 (54.9)		
Non–governmental organization	19 (86.4)	3 (13.6)		
Others	24 (27.0)	65 (73.0)		
Experienced any adverse effects following COVID–19 vaccination				
No	198 (47.9)	215 (52.1)	9.60 (1)	0.002*
Yes	132 (36.9)	226 (63.1)		
Advised other people to get vaccinated for COVID–19				
No	177 (58.4)	126 (41.6)	10.92 (1)	0.001*
Yes	324 (47.0)	365 (53.0)		
The main source of information about the COVID–19 vaccines				
Friends and relatives	80 (73.4)	29 (26.6)	199.25 (4)	< 0.001*
Government owned media	172 (74.5)	59 (25.5)		
Scientific and medical websites	83 (24.5)	256 (75.5)		
Social media platforms	92 (71.9)	36 (28.1)		
Have no information	74 (40.0)	111 (60.0)		
Believing that COVID–19 vaccines could affect reproduction				
No	210 (50.0)	210 (50.0)	2.88 (1)	0.237
Yes	74 (57.4)	55 (42.6)		

*DF* Degrees of freedom

*Statistically significant

**Fig. 2:**
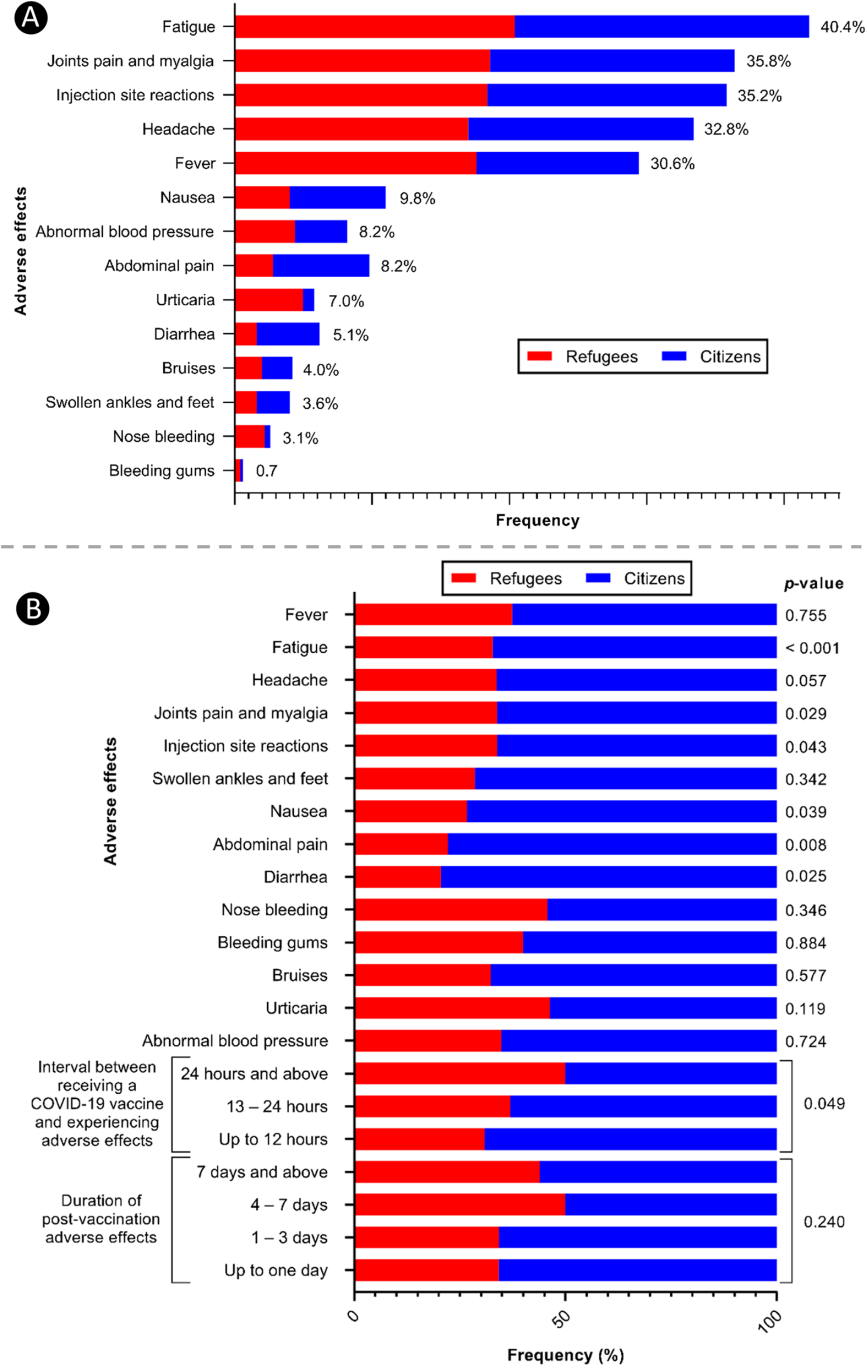
Self-reported adverse effects of COVID-19 vaccines in refugees and citizens. **A** Frequencies of post-vaccination adverse effects among all the vaccinated participants (*n* = 769). **B** Determining of the significant differences in the frequencies of post-vaccination adverse effects between refugees (*n* = 328) and citizens (*n* = 441). A *P*-value ≤ 0.05 is statistically significant

The most frequently reported post-vaccination adverse effects among all participants were general fatigue (40.4%), joint pain and myalgia (35.8%), injection site reactions (35.2%), headache (32.8%) and fever (30.6%) (Fig. 2A). There were significant differences in the frequency of general fatigue (*P* < 0.001), joint pan and myalgia (*P* < 0.029), injection site reactions (*P* < 0.043), nausea (*P* < 0.039), abdominal pain (*P* < 0.008) and diarrhea (*P* < 0.025) between refugees and citizens (Fig. 2B).

Some of the post-vaccination adverse effects (i.e., fever, joints pain and myalgia, and injection site pain or swelling) and the period of adverse effects after vaccination were varied depending on the type of vaccine (Additional file 3: Table S1). However, the proportion of people who received Pfizer-BioNTech is much higher than that of people who received other vaccines. Besides, there was no differences in the frequency of various adverse effects following the first and second doses, except bleeding gum, but the proportion of people who received the second dose is much higher (Additional file 3: Table S2).

### Attitudes towards COVID-19

**Fig. 3:**
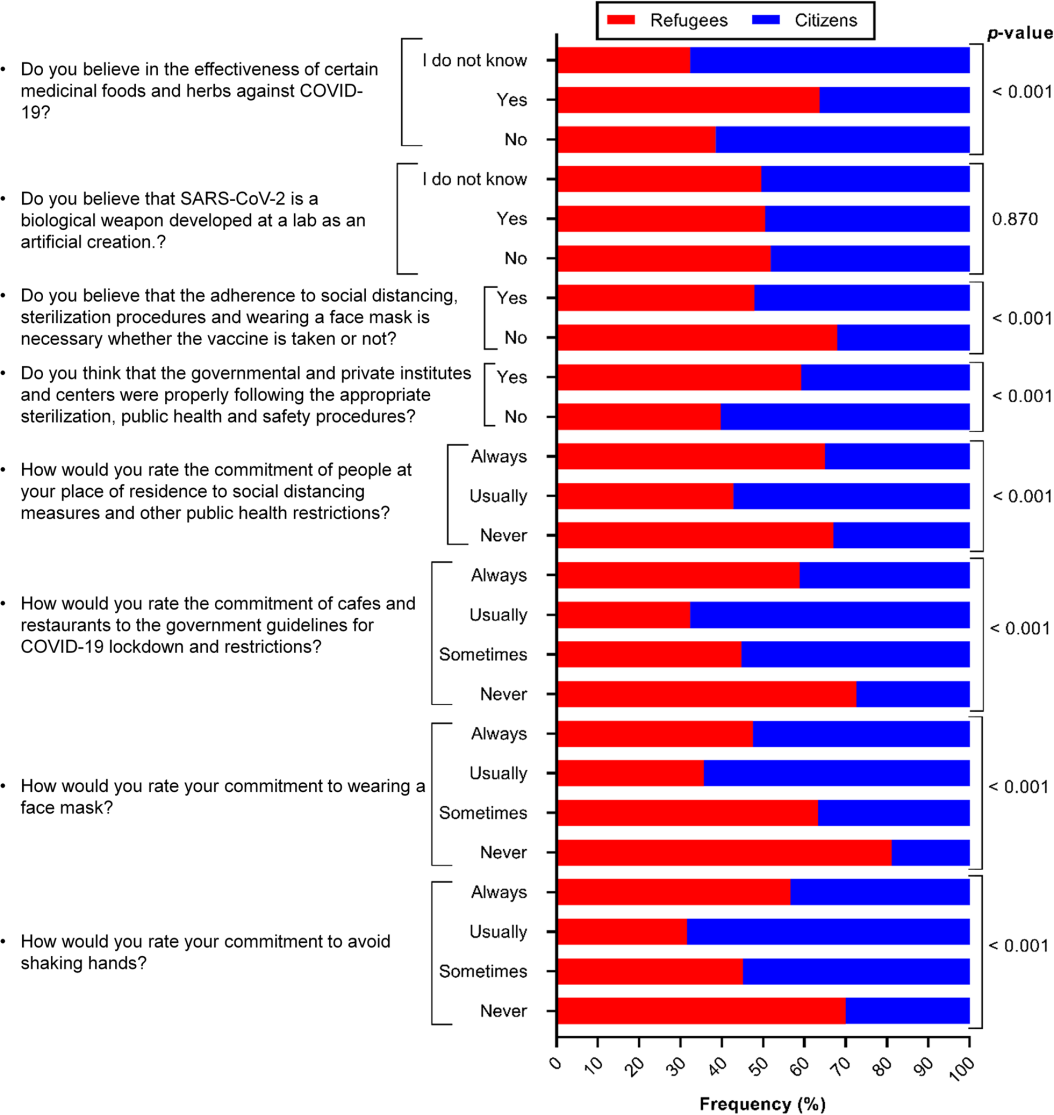
The participants’ perceptions towards COVID-19 infection, lockdown and prevention (*n* = 501 refugees and 491 citizens). A *P*-value ≤ 0.05 is statistically significant

The participants were also asked to answer general questions to evaluate their attitudes towards COVID-19 and its related measures (Fig. 3). Significant differences were reported between refugees’ and citizens’ beliefs about the effectiveness of medicinal plants in combating COVID-19 (*P* < 0.001), and about the need for wearing face masks, practicing social distancing and following proper prevention hygiene among vaccinated people (*P* < 0.001). Their personal commitment to wear a face mask and to avoid shaking hands and their opinions regarding the commitment of restaurants and cafes to follow the precautionary measures were significantly different as well (*P* < 0.001).

### Factors associated with selected variables

From the results in Table 5, three variables were retained in the final model (i.e., gender, age and educational level). For gender, the males were 47% less likely to experience COVID-19 vaccine hesitancy compared to the females (A*OR* = 0.53, *P* < 0.001). For the age group, compared to those who aged 18–30 years, the participants who aged 31–50 years old were 96% more likely to experience COVID-19 vaccine hesitancy (A*OR* = 1.96, *P* < 0.001), the 51–70 years old were 64% more likely (A*OR* = 1.64, *P* = 0.018), and those above 70 years were 67% less likely (A*OR* = 0.33, *P* = 0.166). For the educational level, compared to those with high school level, those who study or obtained their undergraduate degrees were 27% more likely to experience COVID-19 vaccine hesitancy (A*OR* = 1.27, *P* = 0.101), and those who pursued their postgraduate education were 3.2 times more likely to experience COVID-19 vaccine hesitancy (A*OR* = 3.15, *P* = 0.001) [H-L *P* = 0.375; no MC; AUC = 64.4% (Additional file 4: Fig. S1)].

**Table 5:** Results of the logistic regression model assessing factors for COVID-19 vaccine hesitancy (*n* = 992)

Variable	C*OR* (95% *CI*)	*P*–value	A*OR* (95% *CI*)	*P*–value
Residency status				
Refugee	1			
Citizen	1.50 (1.17–1.93)	0.002		
Gender				
Female	1		1	
Male	0.49 (0.38–0.63)	< 0.001	0.53 (0.40–0.69)	< 0.001
Age (years)				
18–30	1		1	
31–50	1.95 (1.48–2.57)	< 0.001	1.96 (1.45–2.64)	< 0.001
51–70	1.40 (0.95–2.07)	0.091	1.64 (1.09–2.47)	0.018
> 70	0.27 (0.06–1.31)	0.104	0.33 (0.07–1.59)	0.166
Educational level				
High school or below	1		1	
Undergraduate	1.19 (0.92–1.54)	0.186	1.27 (0.95–1.69)	0.101
Postgraduate	4.08 (2.17–7.68)	< 0.001	3.15 (1.65–6.02)	0.001
Health insurance				
No	1			
Yes	1.30 (1.01–1.67)	0.041		
Job status				
No	1			
Yes	1.21 (0.93–1.56)	0.151		
Smoking status				
No	1			
Yes	0.64 (0.49–0.82)	0.001		
Food and/or drug allergies				
No	1			
Yes	0.99 (0.68–1.46)	0.975		

Smoking includes all forms of smoking, such as cigar smoking, cigarette smoking, and pipe smoking

*COR* Crude odds ratio, *AOR* Adjusted odds ratio, *CI*: Confidence interval

From the results in Table 6, three variables were retained in the final model (i.e., gender, age, and smoking status). For gender, the males were 56% more likely to believe in the long-term safety of the vaccine compared to the females (A*OR* = 1.56, *P* = 0.004). For the age group, compared to those aged 18–30 years, the participants aged 31–50 years were 47% less likely to believe in the long-term safety of the vaccine (A*OR* = 0.53, *P* < 0.001), the 51–70 years were 55% less likely (A*OR* = 0.45, *P* < 0.001), and those above 70 years were 54% more likely (A*OR* = 1.54, *P* = 0.593). For smoking status, compared to non-smokers, the smokers were 32% less likely to believe in the long-term safety of the vaccine (A*OR* = 0.68, *P* = 0.013) [H-L *P* = 0.001; no MC; AUC = 61.1% (Additional file 4: Fig. S2)].

**Table 6:** Results of the logistic regression model assessing factors for the belief that COVID-19 vaccines are safe in the long-term (*n* = 992)

Variable	C*OR* (95% *CI*)	*P–*value	A*OR* (95% *CI*)	*P*–value
Residency status				
Refugee	1			
Citizen	1.41 (1.10–1.83)	0.008		
Gender				
Female	1		1	
Male	1.28 (0.99–1.66)	0.059	1.56 (1.16–2.11)	0.004
Age (years)				
18–30	1		1	
31–50	0.51 (0.38–0.67)	< 0.001	0.53 (0.40–0.70)	< 0.001
51–70	0.49 (0.33–0.73)	< 0.001	0.45 (0.30–0.68)	< 0.001
> 70	1.78 (0.37–8.50)	0.469	1.54 (0.32–7.40)	0.593
Educational level				
High school or below	1			
Undergraduate	1.49 (1.14–1.95)	0.003		
Postgraduate	0.87 (0.52–1.46)	0.606		
Health insurance				
No	1			
Yes	1.29 (1.00–1.67)	0.050		
Job status				
No	1			
Yes	0.88 (0.68–1.14)	0.324		
Smoking status				
No	1		1	
Yes	0.80 (0.62–1.04)	0.100	0.68 (0.50–0.92)	0.013
Food and/or drug allergies				
No	1			
Yes	1.04 (0.71–1.54)	0.834		

Smoking includes all forms of smoking, such as cigar smoking, cigarette smoking, and pipe smoking

*COR* Crude odds ratio, *AOR* Adjusted odds ratio, *CI* Confidence interval

From the results in Table 7, two variables were retained in the final model (i.e., age and educational level). For age group, compared to those who aged 18–30 years, the 31–50 years old were 44% less likely to believe that COVID-19 vaccines are effective and help in combating the pandemic (A*OR* = 0.66, *P* = 0.005), the 51–70 years were 25% less likely (A*OR* = 0.75, *P* = 0.162), and those above 70 years were 2.6 times more likely (A*OR* = 2.56, *P* = 0.241). For educational level, compared to those with high school level, those with undergraduate degrees were 57% times more likely to believe that COVID-19 vaccines are effective and help in combating the pandemic (A*OR* = 1.57, *P* = 0.002), while those with postgraduate degrees were 3% times more likely (A*OR* = 1.03, *P* = 0.924) [H-L *P* = 0.046; no MC; AUC = 59.5% (Additional file 4: Fig. S3)].

**Table 7:** Results of the logistic regression model assessing factors for the belief that COVID-19 vaccines are effective and help in combating the pandemic (*n* = 992)

Variable	C*OR* (95% *CI*)	*P*–value	A*OR* (95% *CI*)	*P*–value
Residency status				
Refugee	1			
Citizen	1.62 (1.25–2.09)	< 0.001		
Gender				
Female	1			
Male	1.15 (0.89–1.49)	0.280		
Age (years)				
18–30	1		1	
31–50	0.57 (0.43–0.75)	< 0.001*	0.66 (0.49–0.88)	0.005
51–70	0.66 (0.44–0.98)	0.037*	0.75 (0.50–1.12)	0.162
> 70	1.99 (0.42–9.50)	0.387	2.56 (0.53–12.34)	0.241
Educational level				
High school or below	1		1	
Undergraduate	1.73 (1.32–2.26)	0.866	1.57 (1.19–2.08)	0.002
Postgraduate	0.96 (0.57–1.60)	0.089	1.03 (0.61–1.72)	0.924
Health insurance				
No	1			
Yes	1.40 (1.09–1.81)	0.009		
Job status				
No	1			
Yes	0.86 (0.66–1.11)	0.244		
Smoking status				
No	1			
Yes	0.97 (0.75–1.26)	0.815		
Food and/or drug allergies				
No	1			
Yes	1.06 (0.72–1.57)	0.759		

Smoking includes all forms of smoking, such as cigar smoking, cigarette smoking, and pipe smoking

*COR* Crude odds ratio, *AOR* Adjusted odds ratio, *CI* Confidence interval

From the result in Table 8, two variables were retained in the final model (i.e., residency status and gender). For residency status, the citizens were 62% less likely to face difficulties or restrictions upon the registration to receive a COVID-19 vaccine compared to the refugees (A*OR* = 0.38, *P* < 0.001). For gender, the males were 68% more likely to face difficulties or restrictions upon the registration to receive a COVID-19 vaccine compared to the females (A*OR* = 1.68, *P* = 0.012) [H-L *P* = 0.996; no MC; AUC = 65.9% (Additional file 4: Fig. S4)].

**Table 8:** Results of the logistic regression model assessing factors for facing difficulties or restrictions upon the registration to receive a COVID-19 vaccine (*n* = 992)

Variable	C*OR* (95% *CI*)	*P*–value	A*OR* (95% *CI*)	*P*–value
Residency status				
Refugee	1		1	
Citizen	0.33 (0.22–0.50)	< 0.001	0.38 (0.25–0.59)	< 0.001
Gender				
Female	1		1	
Male	2.19 (1.48–3.23)	< 0.001	1.68 (1.12–2.52)	0.012
Age (years)				
18–30	1			
31–50	1.81 (1.18–2.78)	0.007		
51–70	2.23 (1.28–3.89)	0.005		
> 70	4.53 (1.13–18.21)	0.033		
Educational level				
High school or below	1			
Undergraduate	0.40 (0.26–0.60)	< 0.001		
Postgraduate	0.36 (0.14–0.93)	0.035		
Health insurance				
No	1			
Yes	0.61 (0.41–0.89)	0.011		
Job status				
No	1			
Yes	1.20 (0.82–1.75)	0.359		
Smoking status				
No	1			
Yes	1.11 (0.75–1.64)	0.597		
Food and/or drug allergies				
No	1			
Yes	1.46 (0.86–2.45)	0.158		

Smoking includes all forms of smoking, such as cigar smoking, cigarette smoking, and pipe smoking

*COR* Crude odds ratio, *AOR* Adjusted odds ratio, *CI* Confidence interval

From the result in Table 9, two variables were retained in the final model (i.e., age and job status). For age group, compared to those who aged 18–30 years, the participants with 31–50 years old were 96% more likely to believe that SARS-CoV-2 is a biological weapon developed at a lab as an artificial creation (A*OR* = 1.96, *P* < 0.001), the 51–70 years were 9% more likely (A*OR* = 1.09, *P* = 0.736), and those above 70 years were 44% less likely (A*OR* = 0.66, *P* = 0.616). For job status, people who are already employed were 35% more likely to believe that SARS-CoV-2 is a biological weapon developed at a lab as an artificial creation compared to those without a job (A*OR* = 1.35, *P* = 0.085) [H-L *P* = 0.996; no MC; AUC = 60.5% (Additional file 4: Fig. S5)].

**Table 9:** Results of the logistic regression model assessing factors for the belief that SARS-CoV-2 is a biological weapon developed at a lab as an artificial creation (*n* = 992)

Variable	C*OR* (95% *CI*)	*P*–value	A*OR* (95% *CI*)	*P*–value
Residency status				
Refugee	1			
Citizen	1.06 (0.77–1.45)	0.746		
Gender				
Female	1			
Male	0.80 (0.58–1.10)	0.163		
Age (years)				
18–30	1		1	
31–50	2.12 (1.48–3.05)	< 0.001	1.96 (1.35–2.85)	< 0.001
51–70	1.15 (0.71–1.87)	0.568	1.09 (0.67–1.78)	0.736
> 70	0.60 (0.12–3.04)	0.541	0.66 (0.13–3.34)	0.616
Educational level				
High school or below	1		1	
Undergraduate	1.06 (0.76–1.48)	0.721	1.35 (0.96–1.91)	0.085
Postgraduate	1.25 (0.64–2.42)	0.511		
Health insurance				
No	1			
Yes	1.11 (0.81–1.54)	0.514		
Job status				
No	1			
Yes	1.58 (1.14–2.20)	0.007		
Smoking status				
No	1			
Yes	1.34 (0.96–1.87)	0.089		
Food and/or drug allergies				
No	1			
Yes	1.43 (0.85–2.41)	0.177		

Smoking includes all forms of smoking, such as cigar smoking, cigarette smoking, and pipe smoking

*COR* Crude odds ratio, *AOR* Adjusted odds ratio, *CI *Confidence interval

### Controlling for confounding factors

It appears that the significance of association between place of residence and many variables has not changed. The only changed results were those not flagged with a star in Table 10, which indicate that the significance of association between these variables and the place of residence has changed after controlling for confounding factors. The presence of these confounding factors (i.e., age, gender and education level) before controlling appears to have contributed whether the association is significant or not. Post-COVID-19 vaccination adverse effects like fever, swollen ankles and feet, nosebleed, bleeding gums, bruises, urticaria and abnormal blood pressure became significantly associated with the place of residence after controlling for the confounding factors. Additionally, after controlling for the confounding factors, most self-reported COVID-19 symptoms were found to be strongly associated with the place of residence.

**Table 10:** Mantel-Haenszel χ^2^ results for the association of variable A (place of residence) with variable B after controlling for the confounding factors (education level, age and gender)

Variable B	Education Level		Age		Gender	
	*χ*^2^	*P*-value	*χ* ^2^	*P*-value	*χ* ^2^	*P*-value
Self-reported COVID-19 symptoms						
Dyspnea	11.40	< 0.05	32.33	< 0.05	37.66	< 0.05
Cough	1.56	> 0.05*	7.39	< 0.05	9.53	< 0.05
Sore throat	4.35	< 0.05	32.34	< 0.05	31.35	< 0.05
Abdominal pain	7.77	< 0.05	34.37	< 0.05	42.37	< 0.05
Diarrhea	8.20	< 0.05	44.36	< 0.05	44.51	< 0.05
Nausea	6.17	< 0.05	24.36	< 0.05	29.51	< 0.05
Xerostomia	5.43	< 0.05	17.11	< 0.05	24.77	< 0.05
Tiredness and fatigue	1.53	> 0.05*	1.40	> 0.05*	5.51	< 0.05
Headache	1.41	> 0.05*	4.06	< 0.05	5.46	< 0.05
Anorexia	4.97	< 0.05	18.25	< 0.05	22.82	< 0.05
Dizziness	6.84	< 0.05	28.30	< 0.05	34.32	< 0.05
Fever	5.94	< 0.05	12.91	< 0.05	10.90	< 0.05
Urticaria	17.33	< 0.05	53.55	< 0.05	53.64	< 0.05
Anosmia or ageusia	4.49	< 0.05	14.29	< 0.05	17.96	< 0.05
Used antibiotics	13.11	< 0.05	22.39	< 0.05	36.73	< 0.05
The belief that COVID-19 vaccines are safe in the long-term	1.94	> 0.05	1.69	> 0.05	10.78	< 0.05*
The belief that COVID-19 vaccines are effective and help in combating the pandemic	3.51	> 0.05	8.21	< 0.05*	16.80	< 0.05*
Faced difficulties or restrictions upon the registration to receive a COVID-19 vaccine	9.86	< 0.05*	17.62	< 0.05*	18.99	< 0.05*
COVID-19 vaccine hesitancy	2.82	> 0.05	24.81	< 0.05*	2.29	< 0.05*
Refused to receive a vaccine in the past	2.44	> 0.05	8.41	< 0.05*	8.95	< 0.05*
Received a COVID-19 vaccine	9.98	< 0.05*	72.16	< 0.05*	109.60	< 0.05*
Number of doses	8.94	< 0.05*	16.56	< 0.05*	10.52	< 0.05*
Experienced any adverse effects following COVID-19 vaccination	1.00	> 0.05	0.23	> 0.05	8.55	< 0.05*
Advised other people to get vaccinated for COVID-19	6.73	< 0.05*	5.99	< 0.05*	12.45	< 0.05*
Post-COVID-19 vaccination side effects						
Fever	3.92	< 0.05	7.80	< 0.05	11.95	< 0.05
Fatigue	1.46	> 0.05	4.02	< 0.05*	1.70	> 0.05
Headache	0.29	> 0.05*	0.65	> 0.05*	3.12	> 0.05*
Joints pain and myalgia	0.36	> 0.05	0.00	> 0.05	2.06	> 0.05
Injection site reactions	0.00	> 0.05	2.15	> 0.05	1.34	> 0.05
Swollen ankles and feet	8.27	< 0.05	24.71	< 0.05	27.98	< 0.05
Nausea	5.13	< 0.05*	14.34	< 0.05*	18.81	< 0.05*
Abdominal pain	4.92	< 0.05*	16.23	< 0.05*	16.45	< 0.05*
Diarrhea	5.75	< 0.05*	21.81	< 0.05*	22.21	< 0.05*
Nosebleed	13.21	< 0.05	36.50	< 0.05	34.41	< 0.05
Bleeding gums	11.82	< 0.05	34.67	< 0.05	34.15	< 0.05
Bruises	9.27	< 0.05	28.15	< 0.05	29.01	< 0.05
Urticaria	10.51	< 0.05	34.10	< 0.05	35.06	< 0.05
Abnormal blood pressure	9.05	< 0.05	22.91	< 0.05	25.49	< 0.05
The belief that the adherence to social distancing, sterilization procedures and wearing a face mask is necessary whether the vaccine is taken or not	12.37	< 0.05*	16.56	< 0.05*	6.69	< 0.05*
The belief that the governmental and private institutions and centers were properly following the appropriate sterilization, public health and safety procedures	18.33	< 0.05*	30.31	< 0.05*	40.96	< 0.05*

*P*-value ≤ 0.05 indicates that place of residence is associated with variable B after controlling for the confounding factor. *The *P*-value flagged with a star has not changed after controlling

## Discussion

This study showed that Palestinian refugees in Jerash camp live in conditions that could be considered poor. The refugees have low educational levels, a high smoking prevalence, and an extremely high unemployment rate, as well as a lack of health insurance coverage. Thus, most of refugees use only non-profit healthcare centers owned and operated by the government agencies or non-government organizations (NGOs). These factors may underlie the low COVID-19 testing rate, which is more likely contributing to the low rate of the reported cases among the refugees. Interestingly, refugees who were infected with COVID-19 tend to experience symptoms at a lower severity level (self-reported). Furthermore, the most frequently reported symptoms were general fatigue, headache, fever, anosmia and/or ageusia, and cough, respectively, with no significant differences between refugees and citizens. These symptoms were also reported in many previous studies from different regions [35–37].

Interestingly, the results obtained in this study demonstrated that the perceptions and experiences of COVID-19 vaccination were different between refugees and citizens. Although refugees were less likely than citizens to believe that COVID-19 vaccines are safe in the long-term and that these vaccines are effective in combating the pandemic, COVID-19 vaccine hesitancy was significantly lower among refugees. Despite a higher percentage of refugees had refused to receive a vaccine, other than a COVID-19 vaccine, in the past. The final logistic regression model showed that the belief that the long-term safety of COVID-19 vaccines among all participants was affected by gender, age and smoking, while the belief that COVID-19 vaccines are effective and help in combating the pandemic was affected by age and level of education.

The WHO defined vaccine hesitancy as a reluctance to be vaccinated despite the availability of the vaccines, and the organization in 2019 designated hesitancy to get vaccinated as one of the ten threats that affect the global health [38, 39]. In this study, the low vaccine hesitancy among refugees could be associated with the low level of education among them. The final logistic regression model showed that COVID-19 vaccine hesitancy among all participants was affected by gender, age and level of education. As reported in recent studies, people with a lower education level showed significantly greater willingness to receive a COVID-19 vaccine, while those with a higher education level expressed greater vaccine hesitancy [40, 41]. Chen and associates [40] have also reported significant negative correlations between both participants’ monthly income and age with their willing to be vaccinated against COVID-19.

The low vaccine hesitancy among refugees could also be associated with the variation in the sources of information about the COVID-19 vaccines between refugees and citizens. For example, scientific and medical websites were the main source of information among citizens, while higher percentages of refugees rely on information from social media platforms and from their relatives and friends. In another study, Theocharis and associates [42] indicated a significant role of some social media platforms in the spread and upswing of COVID-19 conspiracy theories. As shown in a study by Bullock and associates [43], vaccine hesitancy can be driven by conspiracy theories, fear, doubt, distrust of scientific expertise, and lack of information. Furthermore, the final logistic regression model showed that, among all the participants, the conspiracy theory which says that SARS-CoV-2 is a biological weapon developed at a lab as an artificial creation was affected by age and education level.

Moreover, difficulties or restrictions upon the registration to receive a COVID-19 vaccine were more common among refugees. Refugee-hosting countries may experience a range of legal and administrative barriers to immunization services, including real, restricted or perceived lack of entitlement to free COVID-19 vaccines or health care in general, and a lack of safe and trusted access points [44]. The final logistic regression model showed that facing difficulties or restrictions upon the registration to receive a COVID-19 vaccine among all participants was affected by residency status (refugee or citizen) and gender. Therefore, the percentages of partially- and fully-vaccinated refugees were significantly lower compared to citizens in this study, and a less percentage of vaccinated refugees have advised other people to get vaccinated against COVID-19.

These findings were expected with the limited education and healthcare facilities, as well as the high rate of poverty in Jerash camp [24]. A previous study has shown that Palestinian refugees in Jordan have a lower health-related quality of life compared to Jordanian citizens [45]. However, there are 24 primary healthcare centers run by the UNRWA in the Palestinian refugee camps in Jordan [46]. The United Nations High Commissioner for Refugees (UNHCR), supported by volunteers from the refugees themselves, has provided the refugees with a small package to cover their transportation to the nearest vaccination center. By correcting all the misinformation about the COVID-19 origin and vaccines, a group of these volunteers is helping in fighting against the rumors that refugees believed in and made them hesitant to take a COVID-19 vaccine [47].

The distribution of different types of COVID-19 vaccines (i.e., AstraZeneca, Pfizer–BioNTech and Sinopharm) were similar in refugees and citizens. The most frequently reported post-vaccination adverse effects among all participants were general fatigue, joint pain and myalgia, injection site reactions, headache and fever, respectively. These adverse effects were in a line with that previously reported [28, 48, 49]. More specifically, in a multinational study involving more than 10,000 participants from all Arab countries, including Jordan and Palestine, these adverse effects were also most frequently reported [29]. Vaccine adverse effects are normal signs indicating the immune system is responding to promote protection to the body against the virus. As observed in most vaccines, adverse effects of COVID-19 vaccines range from mild to moderate flu-like symptoms. According to the CDC, the most common adverse effects following COVID-19 vaccination are injection site pain, redness and swelling, as well as fatigue, headache, muscle pain, chills, fever and nausea [50]. Previous studies showed that adverse effects of the COVID-19 vaccine were observed more in individuals who received the Pfizer and the AstraZeneca vaccines compared to those who received the Sinopharm vaccine [28, 29, 51]. Although, our study showed no significant differences in the frequencies of adverse effects based on the type of COVID-19 vaccine or the number of doses. In the present study, the proportion of refugees who experienced post-vaccination adverse effects was lower and there were significant differences between the frequency of some adverse effects between refugees and citizens. The refugees were less likely to experience fatigue, joint pan and myalgia, injection site reactions, nausea, abdominal pain and diarrhea following COVID-19 vaccination. Furthermore, refugees were more likely to face the post-vaccination adverse effects after 24 h and above of vaccination.

COVID-19 vaccine adverse effects are expected to disappear after a few days from their appearance. Furthermore, they may not be experienced by some people and this is attributed to the way each immune system differs in its response [52]. There was no significant difference in the duration of these adverse effects between refugees and citizens. Interestingly, these findings might be consistent with a known theory that indicates people who are mostly habituated to living in poverty with the lesser hygienic conditions and inadequate or restricted access to health services could have naturally acquired better immunity and more resilient to infection [53].

In this study, there were a few additional questions to assess the refugees’ attitudes towards COVID-19 and its related safety and public measures. The percentage of refugees who believe that medicinal plants (e.g., garlic and ginger) and foods (e.g., honey) can be effective in combating COVID-19 was significantly higher. This is not unique in this study, since different population from different countries including China, India, Morocco, Thailand, Bolivia, Nepal and Peru were found to consuming different medicinal plants in order to fight COVID-19 infection [54–60]. Interestingly, the WHO demonstrated that 80% of the population reside in developing countries use traditional medicine as their main source of medical treatment [61]. Furthermore, the Arab World has been characterized through generations by an abundant inventory of medicinal plant usage [62]. In the 21st century, traditional medicinal plants are still commonly used, especially in communities with high poverty rates like refugee camps, as an affordable healthcare regime. In fact, consuming medicinal plants might be useful for the health and boosting the immunity [63], but it cannot be an alternative approach to combat an extreme global pandemic like COVID-19.

A significantly greater proportion of participants who believe that vaccinated people no longer need to wear face masks, practice social distancing and follow proper prevention hygiene was reported among refugees. The personal commitment to wearing a face mask and not shaking hands was significantly lower among refugees. The participants were also asked about their observations regarding the commitment of restaurants and cafes (in their places of residence) to the government rules during the different phases of the COVID-19 lockdown. There was a significant difference between refugees and citizens indicating a lesser level of commitment by the restaurants and cafes in the refugee camp. Again, all these findings were expected and attributed to the refugees’ low educational levels and the lower level of awareness of COVID-19 in the camp. A study conducted in Germany showed that highly educated persons were more worried about the COVID-19 than their peers with lower levels of education [64]. Since it is well-known that health literacy promotes the commitment of individuals to follow public health measures to prevent infectious diseases and cope with pandemics, Naveed and Shaukat [65] have conducted a study to assess this theory among university students in Pakistan. They reported a positive association indicating that greater health literacy promoted COVID-19 awareness and protective behaviors of participants.

Surprisingly, unlike citizens, a significant percentage of refugees believe that public and private institutions and departments were probably following social distancing rules and other COVID-19 public health measures. We were unable to explain this difference; but it might be because the refugees from Jerash camp have limited access to such official institutions and departments compared to citizens.

Indeed, cross-sectional studies are often conducted on a single population, and comparisons of the prevalence and incidence rates are made with the normal (control) groups in the literature. In the present study, we decided to conduct a comparative cross-sectional study on refugees and citizens because we needed data on the same parameters and questions that we included in the survey tool for the refugees. No study had yet addressed all of the issues we assessed among refugees, and the results of related previous studies were inconsistent for some parameters. Additionally, we have to mention that the effects of the various vaccines and people’s attitudes toward them might fluctuate drastically depending on a variety of other conditions.

The findings of controlling for confounding factors suggest two crucial points. First, depending on place of residence, most of the COVID-19 symptoms and post-COVID-19 vaccination adverse effects differ significantly. Second, if confounding variables like age, gender, and education level are not carefully controlled, they may obscure this effect. These findings strongly suggest that not only attitudes and perceptions, but also pathological and physiological symptoms of the COVID-19 infection and vaccination, may vary between minority groups and the general population. These variations might be explained by variations in immunity and health. Furthermore, these characteristics are challenging to study in such studies, especially given that refugees are reluctant to share their financial, insurance and other information.

On the other hand, recent two years have witnessed a runaway increase in the involvement of promising machine learning (ML) approaches that contribute to the global efforts for tackling the COVID‑19 pandemic [29]. Interestingly, ML algorithms with different data models are widely used for predicting several properties and parameters related to COVID-19, including mortality rate, prevalence and severity of symptoms, incubation period, transmission routes, and control strategies [66, 67]. In the current observational study, the logistic regression model was used only to identify the significant factors associated with COVID-19 parameters. While future studies may also incorporate such advance models into large-scale, multicenter surveys to investigate the appropriate prevention and control strategies with minimum costs, and to ensure the normal operations of human society in refugee camps, not just in Jordan but also abroad.

The findings of this study will be of particular interest to researchers, government sectors and NGOs focused on improving the quality of life among refugees. This study will encourage for further exploration of health needs of refugees, especially those who are hosted in camps located poorer countries. Future studies that emphasis on the importance of equal access to quality education, social support and healthcare services for refugees are necessary to develop a more rigorous and systematic understanding of refugees’ needs. Furthermore, challenges facing refugees and international, national, or private agencies that work in refugee camps during and following the COVID-19 pandemic should be studied. Such studies can help to overcome these challenges, and thus strengthen pandemic preparedness and response systems in refugee camps.

In particular, as the leading agency that provides education, health and other services to Palestinian refugees in the Near East, UNRWA would benefit from the findings of this study to address the refugee challenges arising from the COVID-19 pandemic in Jerash camp. UNRWA may also be in a position to collaborate with local researchers to carry out multiple specialized studies to improve its programs in refugee camps for escaping the era of pandemics.

In closing, this study has some limitations including the inability to study the factors that associated with the acceptance of COVID-19 vaccines among the refugees such as source of information and the role of the social media which can play a significant role in spreading a negative information about COVID-19 vaccination. Further, the relevant data from previous research studies were scarce which hardening the discussion and to find the accurate justification of some results. However, this is the first study assessed the experiences and perceptions of COVID-19 infection and vaccination among refugees which prove that the findings of this study might help government policy-makers and NGOs to plan for better healthcare services, health literacy and awareness programs in Jerash camp and other refugee camps in Jordan. This study could be of interest to these parties as it may help in learning lessons from the COVID-19 pandemic which is the first global pandemic the refugees in Jordan have experienced, while the national and global health systems are suffering under immense and exceptional pressures.

## Conclusions

Although they are the least qualified to handle influxes of refugees, developing countries host the largest number of refugees. Data are currently lacking about refugees’ experiences of COVID-19 infection, testing and vaccination, as well as the reasons for low vaccine uptake among them. The findings of this study indicate the importance of increasing the public health awareness and quality of healthcare services in refugee camps, particularly in Jerash camp. The capacity of healthcare services in the refugee-hosting countries must be improved to meet the demands of both refugees and other residents. Since fighting the COVID-19 pandemic requires a set of measures including high vaccine coverage, tracking the spread of variants and regulating prevention measures and ensuring their proper implementation, it is crucial to raise the awareness of refugees towards COVID-19 infection, testing, preventive measures, and the safety and efficacy of vaccines. Furthermore, more equity of COVID-19 testing and vaccination should be applied among the refugees as basic human rights. The accessibility of COVID-19 testing and vaccines among refugees should be the same as among the citizens without discrimination

## Supplementary Information

**Additional file 1.** Survey tool (English version).

**Additional file 2.** Mantel-Haenszel chi-square test.

**Additional file 3: Table S1. **Assessment of the statistical differences inthe frequency of post-vaccination adverse effects based on the type of COVID-19vaccine. **Table S2. **Assessment of the statistical differences in thefrequency of post-vaccination adverse effects based on the number of doses ofCOVID-19 vaccine.

**Additional file 4: Fig. S1.** ROC curve resulted from the logisticregression model assessing factors for the COVID-19 vaccine hesitancy. **Fig.S2. **ROC curve resulted from the logistic regression model assessing factorsfor the belief that COVID-19 vaccines are safe in the long-term. **Fig.** **S3.**ROC curve resulted from the logistic regression model assessing factors for thebelief that COVID-19 vaccines are effective and help in combating the pandemic. **Fig. S4.** ROC curve resulted from the logistic regression model assessingfactors for facing difficulties or restrictions upon the registration toreceive a COVID-19 vaccine. **Fig. S5.** ROC curve resulted from the logisticregression model assessing factors for the belief that SARS-CoV-2 is a biological weapon developed at a lab as an artificial creation.

## Data Availability

All data generated and analyzed during this study are included in this published article.

## Abbreviations

WHO: World Health Organization
COVID-19: Coronavirus disease 2019
SARS-CoV-2: Severe acute respiratory syndrome coronavirus 2
LICs: Low-income countries
MICs: Middle-income countries
HICs: High-income countries
COVAX: COVID-19 Vaccine Global Access
NGOs: Non-government organizations
UNRWA: United Nations Relief and Works Agency for Palestine Refugees in the Near East
UNHCR: United Nations High Commissioner for Refugees
VIFs: Variance inflation factors, AUC:Under the curve
C*OR*: Crude odds ratio
A*OR*: Adjusted odds ratio
MC: Multicollinearity
ROC: Receiver operating characteristics
*CI*: Confidence interval
