# Supplementary material for: Experiences and perceptions of COVID-19 infection and vaccination among Palestinian refugees in Jerash camp and Jordanian citizens: a comparative cross-sectional study by face-to-face interviews
Source: Infect Dis Poverty. 2022 Dec 13;11:123. doi: 10.1186/s40249-022-01047-y (PMC9744667; doi:10.1186/s40249-022-01047-y)
Supplement: Supplementary file 1 — Additional file 1. Survey tool (English version). [file 40249_2022_1047_MOESM1_ESM.docx]

**Additional file 1: Survey tool (English version)**

**Survey of** **experiences and perceptions of COVID-19 infection and vaccination among Palestinian refugees in Jerash camp and Jordanian citizens**

- This survey aims to study the experiences and perceptions of COVID-19 infection and vaccination among Palestinian refugees in Jerash camp compared to Jordanian citizens.
- This survey targets adults aged ≥ 18 years.
- This survey does not include the names or identifying information of participants.
- This survey will not take you more than 10 minutes to complete.

***Participant consent***

By participating in this study, you are contributing in the awareness-raising efforts about the SARS-CoV-2, its effect on individuals in different societies, and the safety and protection of COVID-19 vaccines. Thus, your contribution will help others to protect themselves and to refuse any false rumors about these vaccines. Please note that the participation in this study is voluntary and you can withdraw at any time.

*After knowing the scope and objective of this study, I agree to complete this survey and I promise to answer all questions credibly.

- Yes
- No

***Participant information***

**1. Gender:**

- Male
- Female

**2. Age category (years):**

- 18-30
- 30-50
- 50-70
- 70 years or above

**3. Educational level:**

- High school or below
- College or undergraduate
- Postgraduate

**4. Residency status:**

- Palestinian refugee (in Jerash camp )
- Jordanian citizen (outside refugee camps)

**5. Do you have a health insurance?**

- Yes
- No

**6. Job status:**

- Employed
- Unemployed

**7. Are you a smoker (cigarettes or shisha)?**

- Yes
- No

**8. Are you suffering from an allergy to any types of foods or medicines?**

- Yes
- No

***COVID-19 testing***

**9. Have you get tested for COVID-19?**

- Yes
- No

**10. If you get tested for COVID-19, has the test been done in a governmental or private healthcare center/lab?**

- A governmental sector (free)
- A private sector (paid)

**12. Have you been tested positive for COVID-19?**

- Yes
- No

**13. If you have been tested positive, how would you rate the severity of COVID-19 symptoms?**

- Severe
- Moderate
- Mild
- I did not feel any symptoms

***Self-reported COVID-19 symptoms (if any)***

**14. Have you felt short of breath (dyspnea)?**

- Yes
- No

**15. Have you experienced a cough?**

- Yes
- No

**16. Have you suffered from tonsillitis?**

- Yes
- No

**17. Have you experienced abdominal pain?**

- Yes
- No

**18. Have you experienced diarrhea?**

- Yes
- No

**19. Have you felt nauseous?**

- Yes
- No

**20. Have you felt dry mouth?**

- Yes
- No

**21. Did you feel general fatigue in your body?**

- Yes
- No

**22. Have you felt a headache?**

- Yes
- No

**23. Have you felt a loss of appetite?**

- Yes
- No

**24. Have you felt a loss of balance (dizziness)?**

- Yes
- No

**25. Did you experience fever?**

- Yes
- No

**26. Have you suffered from a rash?**

- Yes
- No

**27. Have you lost your sense of smell or taste or both?**

- Yes
- No

**28. How long have the symptoms last?**

- 1-2 days
- 3-4 days
- Week
- 8 days or more

**29. Have you used any antibiotics as a treatment for the COVID-19?**

- No
- Yes

***Perceptions and attitudes towards COVID-19 vaccination***

**30. Do you agree with the following statements?**

A) The authorized COVID-19 vaccines are generally safe (Yes/No)

B) The authorized COVID-19 vaccines are useful and protect against infection (Yes/No)

**31. Have you faced difficulties or restrictions upon the registration to receive a COVID-19 vaccine?**

- Yes
- No

**32. Did you feel scared to receive a COVID-19 vaccine before vaccination?**

- Yes
- No

**33. Have you refused any previous vaccinations, other than COVID-19 vaccines, in the past?**

- Yes
- No

**34. Have you received a COVID-19 vaccine?**

- Yes
- No

**35. If you have not received any doses of COVID-19 vaccines, what is the reasons for not getting vaccinated so far?**

- I have been infected with COVID-19 before vaccination and that is enough.
- Not interested, not of my priorities and I don't have time
- Fearing of post-vaccination side effects
- I cannot register or I do not know how to register
- I do not trust COVID-19 vaccines, and I don't trust governments and manufacturers
- I think that the mask and the adherence to safety procedures are enough and there is no need for a vaccine
- Others:………………………………

***COVID-19 Vaccination***

**36. Which type of COVID-19 vaccines have you received?**

- Pfizer-BioNTech
- Sinopharm
- AstrZeneca
- Sputnick V
- Other

**37. How many doses have you received so far?**

- 1 dose
- 2 doses

**38. Have you experienced any side effects following COVID-19 vaccination?**

- Yes
- No

***Post-COVID-19 vaccination side effects (if any)***

**39. Have you felt a fever?**

- Yes
- No

**40. Have you felt tiredness and fatigue?**

- Yes
- No

**41. Have you felt a headache?**

- Yes
- No

**42. Have you felt joint pain?**

- Yes
- No

**43. Have you experienced pain or swelling at the injection site?**

- Yes
- No

**44. Have you experienced swollen ankles and feet?**

- Yes
- No

**45. Have you felt nausea?**

- Yes
- No

**46. Have you felt abdominal pain?**

- Yes
- No

**47. Have you experienced diarrhea?**

- Yes
- No

**48. Have you experienced a nosebleed?**

- Yes
- No

**49. Have you experienced bleeding gums?**

- Yes
- No

**50. Have you noticed any bruises on your body?**

- Yes
- No

**51. Have you experienced irritation and allergic skin reaction, or itchy skin?**

- Yes
- No

**52. Have you experienced an increase or decrease in blood pressure?**

- Yes
- No

**53. How soon did the side effects appear after injection with a COVID-19 vaccine?**

- 5 to 12 hours
- 13 to 24 hours
- 24 hours or more

**54. How long did the side effects last?**

- less than one day
- 1 to 3 days
- 4 to 7 days
- More than 7 days

***General beliefs about COVID-19 infection and vaccines***

**55. Do you advice others to take a COVID-19 vaccine?**

- Yes
- No

**56. What are the most reliable sources for information about COVID-19 vaccines?**

- Official media (television, newspaper, … etc)
- Family, neighbors and friends
- Social media (Facebook, WhatsApp, … etc)
- Medical and scientific websites
- I do not trust any source regarding vaccines

**57. Do you believe in the effectiveness of certain medicinal foods and herbs (e.g., garlic, onion, ginger) against COVID-19?**

- Yes
- No
- I do not know

**58. Do you believe that the SARS-CoV-2 is part of a biological warfare and the virus was artificially made?**

- Yes
- No
- I do not know

**59. Do you believe that the adherence to social distancing, sterilization procedures and wearing a face mask is necessary whether the vaccine is taken or not?**

- Yes
- No

**60. How would do you rate the commitment of people at your place of residence to social distancing measures and other public health restrictions?**

- Always
- Usually
- Sometimes
- Never

**61. Do you think that the governmental and private institutes and centers were properly following the appropriate sterilization, public health and safety procedures?**

- Yes
- No

**62. How would do you rate the commitment of cafes and restaurants to the government guidelines for COVID-19 lockdown and restrictions?**

- Always
- Usually
- Sometimes
- Never

**63. How would do you rate your commitment to wearing a mask?**

- Always
- Usually
- Sometimes
- Never

**64. How would do you rate your commitment to not shaking hands?**

- Always
- Usually
- Sometimes
- Never
