# Supplementary material for: Experiences and perceptions of COVID-19 infection and vaccination among Palestinian refugees in Jerash camp and Jordanian citizens: a comparative cross-sectional study by face-to-face interviews
Source: Infect Dis Poverty. 2022 Dec 13;11:123. doi: 10.1186/s40249-022-01047-y (PMC9744667; doi:10.1186/s40249-022-01047-y)
Supplement: Supplementary file 2 — Additional file 2. Mantel-Haenszel chi-square test. [file 40249_2022_1047_MOESM2_ESM.docx]

**Additional file 2: Mantel-Haenszel chi-square test**

The statistic is computed as:

1. *Null and alternative hypotheses:*

H_0_: Variable A (place of residence) is not associated with variable B after controlling for the confounding factor.

H_1_: Variable A (place of residence) is associated with variable B after controlling for the confounding factor.

1. *Stratum “i” by confounder subcategory:*

| ***Variable B*** | ***Variable A*** | | | |
| --- | --- | --- | --- | --- |
|  |  | *Yes* | *No* |  |
|  | *Yes* | *a_i_* | *b_i_* | *a_i_ + b_i_* |
|  | *No* | *c_i_* | *d_i_* | *c_i_ + d_i_* |
|  |  | *a_i_ + c_i_* | *b_i_ + d_i_* | *a_i_ + b_i_ + c_i_ + d_i_* |

1. *Compute Mantel-Haenszel χ^2^ by:*

$$Mantel Haenszel \chi^{2}=\frac{\left( \left| O-E \right|-0.5 \right)^{2}}{V}$$

which has a χ^2^ (one degree of freedom) under H_0_.

Where;

$$e_{i}=\frac{\left( a_{i}+b_{i} \right)\left( a_{i}+c_{i} \right)}{n_{i}}$$

$$v_{i}=\frac{\left( a_{i}+b_{i} \right)(c_{i}+d_{i})\left( a_{i}+c_{i} \right){(b}_{i}+d_{i})}{\left( {n_{i}}^{2} \right) ({n_{i}}-1)}$$

$$O= \sum a_{i}, E= \sum e_{i}, V= \sum v_{i}$$
