# Supplementary material for: Experiences and perceptions of COVID-19 infection and vaccination among Palestinian refugees in Jerash camp and Jordanian citizens: a comparative cross-sectional study by face-to-face interviews
Source: Infect Dis Poverty. 2022 Dec 13;11:123. doi: 10.1186/s40249-022-01047-y (PMC9744667; doi:10.1186/s40249-022-01047-y)
Supplement: Supplementary file 3 — Additional file 3: Table S1. Assessment of the statistical differences inthe frequency of post-vaccination adverse effects based on the type of COVID-19vaccine. Table S2. Assessment of the statistical differences in thefrequency of post-vaccination adverse effects based on the number of doses ofCOVID-19 vaccine. [file 40249_2022_1047_MOESM3_ESM.docx]

**Additional file 3**

| **Table S1.** Assessment of the statistical differences in the frequency of post-vaccination adverse effects based on the type of COVID-19 vaccine. | | | | | | |
| --- | --- | --- | --- | --- | --- | --- |
| **Adverse effects** | **Type of vaccine** | | | | **Chi-square (DF)** | ***p*-value** |
|  | **AstraZeneca**  ***n* (%)** | **Other**  ***n* (%)** | **Pfizer–BioNTech**  ***n* (%)** | **Sinopharmn**  ***n* (%)** |  |  |
| Fever  No  Yes | 1 (0.8)  21 (9.0) | 1 (0.8)  1 (0.4) | 83 (68.0)  165 (70.8) | 37 (30.3)  46 (19.7) | 12.82 (3) | 0.005* |
| Fatigue  No  Yes | 2 (0)  20 (60.5) | 0 (-)  2 (0.6) | 28 (60.9)  220 (71.2) | 16 (34.8)  67 (21.7) | 4.14 (3) | 0.247 |
| Headache  No  Yes | 4 (3.8)  18 (7.2) | 0 (-)  2 (0.8) | 77 (73.3)  171 (68.4) | 24 (22.9)  59 (23.6) | 2.49 (3) | 0.478 |
| Joints pain and myalgia  No  Yes | 4 (4.9)  18 (6.6) | 2 (2.4)  0 (-) | 48 (58.5)  200 (73.3) | 28 (34.1)  55 (20.1) | 14.20 (3) | 0.003* |
| Pain or swelling at the injection site  No  Yes | 8 (9.3)  14 (5.2) | 0 (-)  2 (0.7) | 43 (50.0)  205 (76.2) | 35 (40.7)  48 (17.8) | 23.37 (3) | < 0.001* |
| Swollen ankles and feet  No  Yes | 22 (6.7)  0 (-) | 1 (0.3)  1 (3.6) | 229 (70.0)  19 (67.9) | 75 (22.9)  8 (28.6) | 7.13 (3) | 0.068 |
| Nausea  No  Yes | 17 (6.0)  5 (6.8) | 1 (0.4)  1 (1.4) | 199 (70.8)  49 (66.2) | 64 (22.8)  19 (25.7) | 1.47 (3) | 0.690 |
| Abdominal pain  No  Yes | 19 (6.5)  3 (4.8) | 1 (0.3)  1 (1.6) | 207 (70.6)  41 (66.1) | 66 (22.5)  17 (27.4) | 2.37 (3) | 0.500 |
| Diarrhea  No  Yes | 21 (6.6)  1 (2.6) | 2 (0.6)  0 (-) | 223 (70.6)  25 (64.1) | 70 (22.2)  13 (33.3) | 3.24 (3) | 0.355 |
| Nose bleeding  No  Yes | 22 (6.6)  0 (-) | 2 (0.6)  0 (-) | 226 (68.3)  22 (91.7) | 81 (24.5)  2 (8.3) | 5.99 (3) | 0.112 |
| Bleeding gums  No  Yes | 22 (6.3)  0 (-) | 2 (0.6)  0 (-) | 245 (70.0)  3 (60.0) | 81 (23.1)  2 (40.0) | 1.01 (3) | 0.798 |
| Any bruises on your body  No  Yes | 21 (6.5)  1 (3.2) | 2 (0.6)  0 (-) | 230 (71.0)  18 (58.1) | 71 (21.9)  12 (38.7) | 4.77 (3) | 0.190 |
| Irritation and allergic skin reactions  No  Yes | 21 (7.0)  1 (1.9) | 2 (0.7)  0 (-) | 214 (71.1)  34 (63.0) | 64 (21.3)  19 (35.2) | 6.53 (3) | 0.089 |
| Abnormal blood pressure  No  Yes | 17 (5.8)  5 (7.9) | 2 (0.7)  0 (-) | 208 (71.2)  40 (63.5) | 65 (22.3)  18 (28.6) | 2.13 (3) | 0.545 |
| How long side effects appear  13 – 24 hours  5 – 12 hours  24 hours and above | 12 (9.9)  1 (1.8)  9 (5.1) | 1 (0.8)  0 (-)  1 (0.6) | 87 (71.9)  33 (58.9)  128 (71.9) | 21 (17.4)  22 (39.3)  40 (22.5) | 14.44 (6) | 0.025 |
| How long side effects last  Up to one day  1 – 3 days  4 – 7 days  7 days and above | 3 (3.9)  16 (7.9)  3 (8.8)  0 (-0 | 1 (1.3)  0 (-)  1 (2.9)  0 (-) | 51 (66.2)  147 (72.4)  23 (67.6)  27 (65.9) | 22 (28.6)  40 (19.7)  7 (20.6)  14 (34.1) | 14.69 (9) | 0.100 |

*Statistically significant

| **Table S2.** Assessment of the statistical differences in the frequency of post-vaccination adverse effects based on the number of doses of COVID-19 vaccine. | | | | |
| --- | --- | --- | --- | --- |
| **Adverse effects** | **Number of doses** | | **Chi-square (DF)** | ***p*-value** |
|  | **One**  ***n* (%)** | **Two**  ***n* (%)** |  |  |
| Fever  No  Yes | 12 (9.8)  18 (7.7) | 110 (90.2)  217 (92.3) | 0.49 (1) | 0.482 |
| Fatigue  No  Yes | 7 (15.2)  23 (7.4) | 39 (84.8)  288 (92.6) | 3.19 (1) | 0.074 |
| Headache  No  Yes | 10 (9.5)  20 (7.9) | 95 (90.5)  232 (92.1) | 0.24 (1) | 0.622 |
| Joints pain and myalgia  No  Yes | 8 (9.8)  22 (8.0) | 74 (90.2)  253 (92.0) | 0.24 (10) | 0.615 |
| Pain or swelling at the injection site  No  Yes | 8 (9.3)  22 (8.1) | 78 (90.7)  249 (91.9) | 0.12 (1) | 0.730 |
| Swollen ankles and feet  No  Yes | 27 (8.2)  3 (10.7) | 302 (91.8)  25 (89.3) | 0.21 (1) | 0.646 |
| Nausea  No  Yes | 22 (7.8)  8 (10.7) | 260 (92.2)  67 (89.3) | 0.63 (1) | 0.427 |
| Abdominal pain  No  Yes | 24 (8.2)  6 (9.5) | 270 (91.8)  57 (90.5) | 0.13 (1) | 0.724 |
| Diarrhea  No  Yes | 27 (8.5)  3 (7.7) | 291 (91.5)  36 (92.3) | 0.03 (1) | 0.865 |
| Nose bleeding  No  Yes | 27 (8.1)  3 (12.5) | 306 (91.9)  21 (87.5) | 0.56 (1) | 0.454 |
| Bleeding gums  No  Yes | 28 (8.0)  2 (40.0) | 324 (92.0)  3 (60.0) | 6.58 (1) | 0.010* |
| Any bruises on your body  No  Yes | 27 (8.3)  3 (9.7) | 299 (91.7)  28 (90.3) | 0.07 (1) | 0.789 |
| Irritation and allergic skin reactions  No  Yes | 25 (8.3)  5 (9.3) | 278 (91.7)  49 (90.7) | 0.06 (1) | 0.806 |
| Abnormal blood pressure  No  Yes | 23 (7.8)  7 (11.1) | 271 (92.2)  56 (88.9) | 0.73 (1) | 0.393 |
| How long side effects appear  13 – 24 hours  5 – 12 hours  24 hours and above | 5 (4.1)  5 (8.9)  20 (11.2) | 118 (95.9)  51 (91.1)  158 (88.8) | 4.88 (2) | 0.087 |
| How long side effects last  Up to one day  1 – 3 days  4 – 7 days  7 days and above | 7 (9.0)  18 (8.8)  3 (8.8)  2 (4.9) | 71 (91.0)  186 (91.2)  31 (91.2)  39 (95.1) | 0.75 (3) | 0.861 |

*Statistically significant
