# Supplementary material for: Experiences and perceptions of COVID-19 infection and vaccination among Palestinian refugees in Jerash camp and Jordanian citizens: a comparative cross-sectional study by face-to-face interviews
Source: Infect Dis Poverty. 2022 Dec 13;11:123. doi: 10.1186/s40249-022-01047-y (PMC9744667; doi:10.1186/s40249-022-01047-y)
Supplement: Supplementary file 4 — Additional file 4: Fig. S1. ROC curve resulted from the logisticregression model assessing factors for the COVID-19 vaccine hesitancy. Fig.S2. ROC curve resulted from the logistic regression model assessing factorsfor the belief that COVID-19 vaccines are safe in the long-term. Fig. S3.ROC curve resulted from the logistic regression model assessing factors for thebelief that COVID-19 vaccines are effective and help in combating the pandemic. Fig. S4. ROC curve resulted from the logistic regression model assessingfactors for facing difficulties or restrictions upon the registration toreceive a COVID-19 vaccine. Fig. S5. ROC curve resulted from the logisticregression model assessing factors for the belief that SARS-CoV-2 is a biological weapon developed at a lab as an artificial creation. [file 40249_2022_1047_MOESM4_ESM.docx]

**Additional file 4**


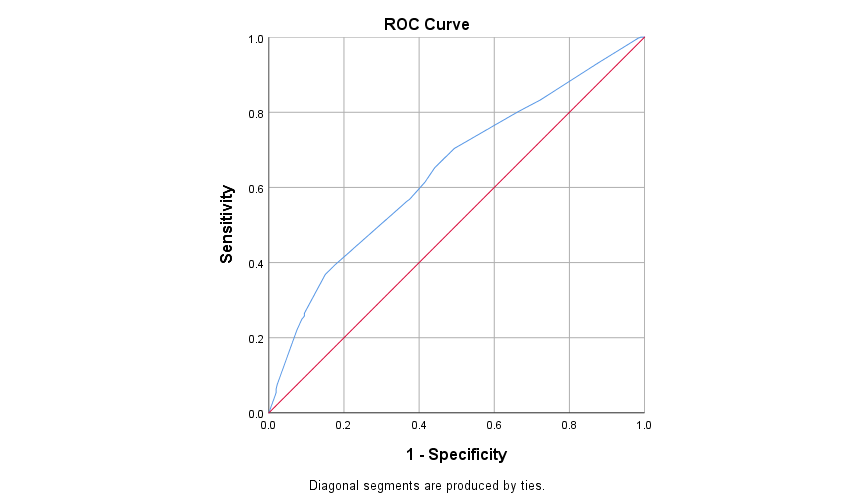


**Fig. S1.** ROC curve resulted from the logistic regression model assessing factors for the COVID-19 vaccine hesitancy.


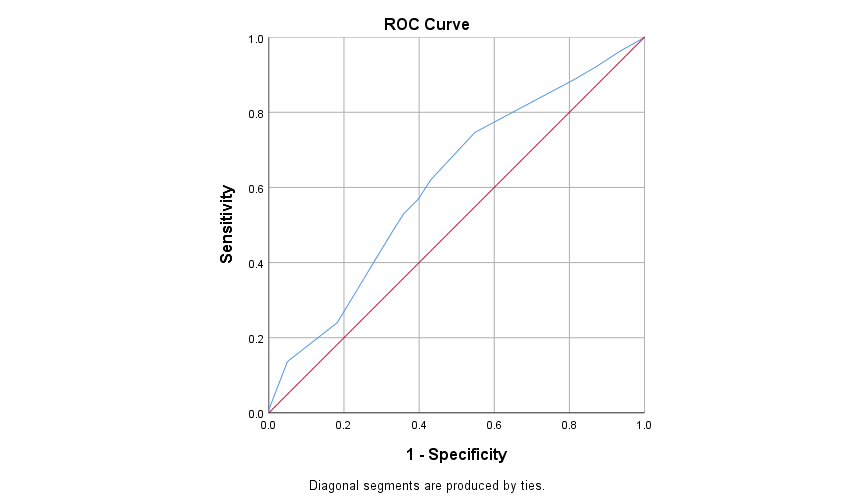


**Fig. S2.** ROC curve resulted from the logistic regression model assessing factors for the belief that COVID-19 vaccines are safe in the long-term.


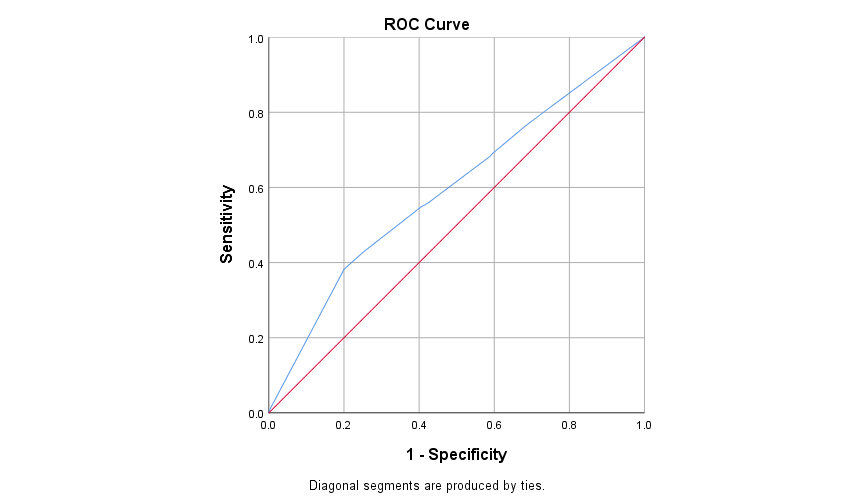


**Fig.** S**3.** ROC curve resulted from the logistic regression model assessing factors for the belief that COVID-19 vaccines are effective and help in combating the pandemic.


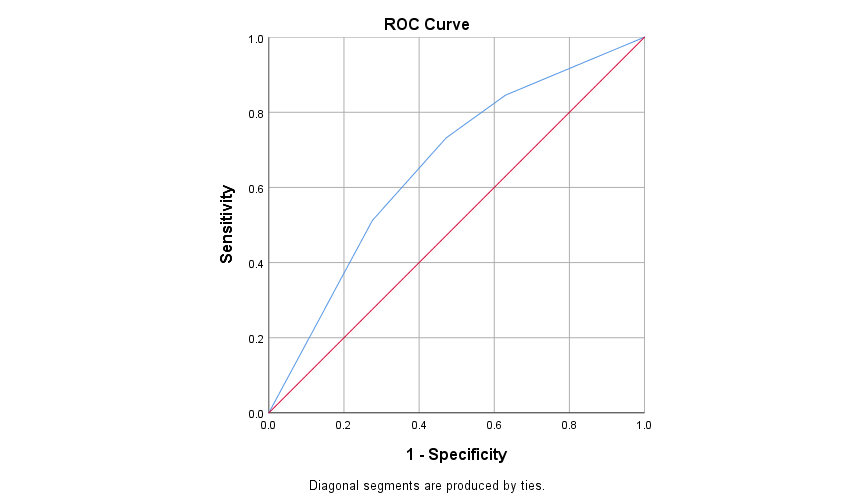


**Fig. S4.** ROC curve resulted from the logistic regression model assessing factors for facing difficulties or restrictions upon the registration to receive a COVID-19 vaccine.


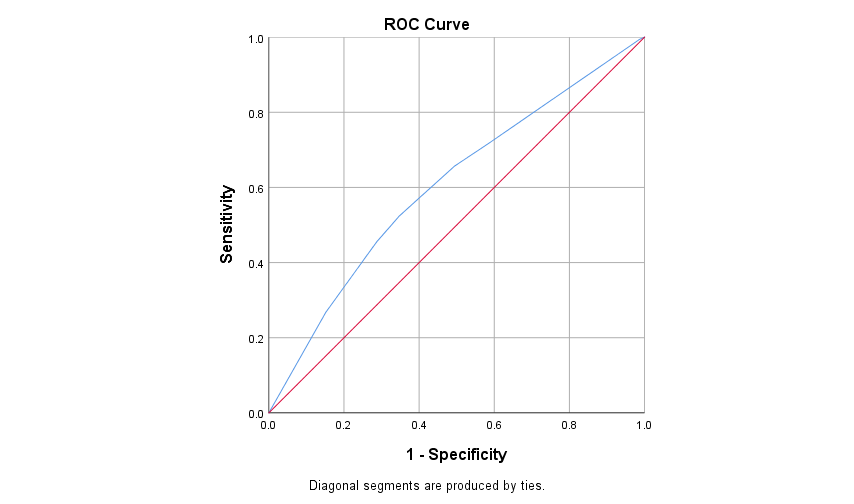


**Fig. S5.** ROC curve resulted from the logistic regression model assessing factors for the belief that SARS-CoV-2 is a biological weapon developed at a lab as an artificial creation.
